# Early prognosis prediction in acute myeloid and acute lymphoid leukemia patients using cell-free DNA concentration ratios

**DOI:** 10.3389/fmolb.2023.1333943

**Published:** 2024-01-22

**Authors:** Noreen Grace George, Bhavika Rishi, Amitabh Singh, Sree Vishmaya, Rakesh Kumar, Neetu Kushwaha, Manpreet Kaur, Reena Bhardwaj, Ankur Jain, Aditi Jain, Sumita Chaudhry, Aroonima Misra

**Affiliations:** ^1^ ICMR‐National Institute of Pathology, New Delhi, India; ^2^ Vardhman Mahavir Medical College and Safdarjung Hospital, New Delhi, India

**Keywords:** prognosis, ratio of cell-free DNA at diagnosis/remission acute myeloid leukemia, acute lymphoblastic leukemia, bone marrow, complete remission

## Abstract

**Background:** Cell-free DNA (cfDNA) is a promising biomarker for disease prediction in many cancers, including acute leukemia (acute myeloid leukemia [AML] and acute lymphoblastic leukemia [ALL]). This study investigated the role of cfDNA in predicting relapse or unfavorable outcomes in acute leukemia patients upon initial diagnosis.

**Methods:** Paired peripheral blood samples of 25 patients with ALL and AML were compared at baseline and induction/follow-up and clinically correlated with clinicopathological and outcome variables according to the risk category. cfDNA was isolated using commercial cfDNA extraction kits. The probability of poor outcomes in high-risk groups and a cut-off value for risk stratification minimal residual disease (MRD) positivity and outcome prediction were derived.

**Results:** Twenty-five patients diagnosed with AML and ALL were risk-stratified based on NCI risk stratification, and of these 25 patients, 4 patients were of standard risk (SR) and 1 patient was of intermediate risk (IR), while a majority of patients (80%) were of high risk (HR). Of these, four HR patients passed away. The ratio of cfDNA reduction at baseline and the end of induction was a strong predictor of poor outcomes in high-risk patients, regardless of the MRD status. A cfDNA ratio score of 2.6 or higher at diagnosis/remission predicted poor outcomes, with higher accuracy than conventional MRD detection by flow cytometry.

**Conclusion:** A higher cfDNA ratio at diagnosis/remission or at baseline predicts poor outcomes in acute leukemia patients. This pilot study suggests that cfDNA ratio scoring may be a useful tool for predicting prognosis in acute leukemia patients, regardless of the MRD status.

## Introduction

Leukemia, the most common cause of pediatric malignancy and also one of the top cancers in adults, is characterized by the uncontrolled proliferation of abnormal white blood cells in the bone marrow (BM) and blood and represents a significant challenge in the field of oncology. Despite considerable advancements in our understanding of the molecular and genetic basis of leukemia, as well as the development of innovative therapeutic strategies, the disease continues to pose a clinical and research challenge globally. In view of heterogeneous etiology and poor prognostic outcomes, new predictive biomarkers are constantly being researched for leukemia (Alaggio et al., 2022).

Circulating free DNA (cfDNA), also known as cell-free DNA or liquid biopsy, represents a revolutionary paradigm in the diagnosis and monitoring of leukemia. Cell-free DNA consists of short, fragmented DNA molecules that are released into the bloodstream by apoptotic or necrotic cells, including leukemic cells (Yan et al., 2021; Ruan et al., 2021). The amount of circulating tumor DNA (ctDNA) found in cfDNA can vary greatly, from 0.01% to 60% of the tumor percentage. This variation depends on several factors related to the tumor such as its size ((larger tumors have more chances of having cfDNA in peripheral blood), stage, blood vessel growth, growth rate, later stages of tumor where angiogenesis is more, and the fragments and tumor cells undergoing apoptosis dependent also on the tumor growth; these tumors have a higher fraction and incidence of cfDNA in peripheral blood (Li et al., 2017; Martignano, 2019; Wu et al., 2019).

The advent of DNA sequencing, particularly the emergence and wider availability and cost-effective use of next-generation sequencing (NGS) technologies, has led to the enhanced utility of cfDNA due to the identification of various genetic mutations associated with malignancies (Barault et al., 2018; Alaggio et al., 2022; Allegra et al., 2022).^.^ cfDNA has shown potential in the realm of leukemia and solid tumors (Yan et al., 2021). The applicability of cfDNA in the prediction of prognosis is another avenue that has been extensively studied in solid tumors with excellent correlation to tissue samples. Many studies have reported that baseline cfDNA concentrations prove to be valuable in the prediction of prognosis in solid tumors, especially renal, breast, genitourinary, and brain tumors (De Mattos et al., 2011; Telekes and Horváth, 2022). The recent World Health Organization (WHO) classification update underscores the presence of recurrent mutations in several hematological malignancies (Alaggio et al., 2022). Several studies have highlighted cfDNA analysis as biomarkers in the monitoring of patients with multiple myeloma (Ntanasis-Stathopoulos et al., 2020; Allegra et al., 2022)**.** In the context of leukemia, Yegin et al. (2020) strikingly demonstrated that AML patients with subsequent post-transplant relapse exhibited notably lower pre-transplant cfDNA levels compared to those who remained in remission.

The application of cell-free DNA encounters several challenges (Lim et al., 2021). Unlike solid tumurs, leukemia often involves blood and bone marrow, making it more challenging to isolate disease-specific cfDNA from the background of normal hematopoietic DNA (Ruan et al., 2021). In addition, the limited number of mutations and genetic variation in leukemia can restrict the effectiveness of cfDNA analysis. That means that the true mutational representation of tumor is difficult in the cfDNA fraction because not all clones shall harbor the mutation-like myeloid lymphoblastic leukemia (MLL) rearrangement or RUNX1 fusions that are present in blasts only and not the differentiated cells (Bohers et al., 2021). Wong et al. (2018) highlighted these issues in acute myeloid leukemia (AML), where the detection of actionable mutations using cfDNA was less reliable than in solid tumors. Additionally, the rapid turnover of leukemia cells can lead to fluctuations in cfDNA levels, posing challenges for consistent monitoring (Punnoose et al., 2012; Ruan et al., 2021). These challenges necessitate on-going research to refine cfDNA-based approaches in leukemia management.

The current use of cfDNA in clinical practice is limited to prenatal testing, detecting chromosomal abnormalities (Esposito et al., 2017). A few cases for transplantation monitoring utilize cfDNA to detect organ rejection and diagnose genetic disorders, such as Rhesus D incompatibility during pregnancy and neonatal care, and infectious diseases. Despite its multifaceted utility, the broader integration of cfDNA into clinical practice faces notable challenges (Stawski et al., 2021; Nikanjam et al., 2022)^.^

Current methods to detect relapse and diagnose minimal residual disease (MRD) rely on invasive bone marrow samples. The development of more sensitive assays can prove to be a valuable tool for monitoring disease progression as a real-time assessment that can guide chemotherapy decisions, leading to more personalized and effective therapeutic interventions. cfDNA-based MRD assessment is emerging as a powerful tool to identify the presence of residual leukemic cells after treatment, even when conventional methods indicate remission so that timely risk-adapted therapeutic intervention can be initiated to prevent relapse.

However, there are serious challenges that need addressing before this attractive revolutionizing technology is applicable and translated into clinical practice in cancers. The clinical application of cfDNA in diagnostic tests faces several challenges across the analytical, pre-analytical, and post-analytical phases, hampering its widespread adoption, as discussed later in this paper (Leon et al., 1977; Li et al., 2017; Greytak et al., 2020; Krasic et al., 2021; Lim et al., 2021; Venetis et al., 2023).

Extensive research on cfDNA is currently being conducted in leukemia patients as it is an attractive alternative for a painful bone marrow biopsy for disease prognostication and monitoring in leukemia. Although a few sequencing studies have been conducted particularly in myeloma and lymphoma on cfDNA, its role in the prognosis and monitoring of disease is yet to be established. To date, there has been a scarcity of research examining the potential of cfDNA, which is a relatively non-invasive source, for monitoring leukemia-related mutations and offering prognostic insights in individuals with hematologic malignancies, without the use of an expensive and labor-intensive methodology (sequencing or other molecular techniques) (Greytak et al., 2020). Nevertheless, in a resource constraint setting, where routine sequencing and access to NGS capabilities are restricted, the adoption of labor-intensive, resource-demanding, and technically intricate tests for leukemia appears impractical and unadvisable. Therefore, it is imperative to explore and seek superior alternatives for the processing and utilization of cfDNA in leukemia.

Many researchers suggested that higher cfDNA ratios lead to poor prognosis of solid tumors. Numerous studies have indeed proposed a correlation between elevated cfDNA ratios and adverse prognoses in solid tumors (Rogers et al., 2004; Mueller et al., 2006; Schwarzenbach et al., 2011; Punnoose et al., 2012; Spindler et al., 2012; Lu and Liang, 2016; Pérez-Ramírez et al., 2016; Phallen et al., 2017; Martignano, 2019; Ntanasis-Stathopoulos et al., 2020; Ruan et al., 2021; Nikanjam et al., 2022; Medina et al., 2023). For instance, Barault et al. (2018) found that a higher cfDNA ratio, specifically the ratio of tumor-derived to non-tumor cfDNA, was associated with advanced disease stages and poorer survival outcomes in colorectal cancer patients. Similarly, in breast cancer, Cheng et al. (2018) demonstrated that an increased tumor cfDNA fraction was linked to worse progression-free survival. These findings underscore the potential of cfDNA ratios as prognostic indicators in solid tumors, emphasizing their clinical relevance in assessing disease severity and guiding treatment decisions (Punnoose et al., 2012; Phallen et al., 2017). To date, apart from target identification in cfDNA for leukemia, no published research study has undertaken a comparative analysis of cfDNA ratios at baseline and follow-up stages to ascertain the prognostic implications for patients with leukemia. The utilization of straightforward cfDNA ratios as a means of defining clinical outcomes has the potential to present a highly practical and cost-efficient approach for prognostication and monitoring in cases of acute leukemia.

## Materials and methods

1) Patients and samples:

Based on the prior published work, researchers investigated the baseline concentration of cfDNA in acute leukemia patients. Twenty-five patients were diagnosed with AML and ALL based on molecular and flow cytometric typing (Table 1 highlights the patient baseline characteristics). Paired peripheral blood /bone marrow samples were collected at baseline and induction/follow-up and clinically correlated with outcome variables. The samples were collected at two end points after ethical consent and institutional review board clearance.

2) Plasma, cfDNA, and clinical data:

**TABLE 1:** Baseline characteristics of the study population (N = 25).

Age (years)	Median	Range
	5	1.5–13
Gender	Number	Percentage
Female	12	48.0
Male	13	52.0
Cytogenetics		
t (15; 17) (q24; q21)	2	8.0
del (1) (p32)	2	8.0
t (9; 22) (q34; q11)	3	12.0
t (12; 21) (p13; q22)	3	12.0
Negative	14	56.0
Alive	21	84.0
Expired	3	12.0
Not contactable	1	4.0
Relapse	3	12.0

Twenty-five pediatric patients diagnosed with acute leukemia were evaluated, and this table shows the baseline characteristics of the study cohort.

The samples were collected immediately following diagnosis using flow cytometry. Clinicopathological variables, such as presentation, laboratory parameters, and clinical factors such as age and sex, were collected to calculate and record the respective NCI risk. Consent was obtained from all enrolled patients, and 4 mL blood samples were collected at two time points: diagnosis and follow-up. The samples were transported in Streck tubes within 30 min, and cfDNA was extracted within 4 h of collection. A QIAGEN QIAamp Circulating Nucleic Acid Kit, along with a vacuum pump-based approach, was utilized for the extraction of cfDNA from peripheral blood samples of AML and ALL patients. The tubes were labeled with unique barcodes and stored at 4°C until further processing, avoiding freezing. Plasma separation was performed by centrifuging samples at 1,600x g 10 min at 4°C. The plasma was then transferred to sterilized tubes to remove cellular matter. cfDNA was extracted using the QIAGEN QIAamp Circulating Nucleic Acid Kit protocol. The plasma, lysis buffer, and carrier RNA were incubated at 56°C and then added to the QIAamp Mini spin column, which is attached to the vacuum pump for extraction and purification. The cfDNA was eluted in 50–100 µL using the provided elution buffer. Quality control measures were implemented, and samples were stored at −80°C until further analysis.

## Determination of cfDNA levels

A standard reference cut-off was decided based on published values of cfDNA threshold—180 ng/mL—based on the published literature for average values of cfDNA (Phallen et al., 2017). This was used to categorize the patients into two groups—low-baseline cfDNA group and high-baseline cfDNA group. Their outcomes were correlated with the risk criteria, and prediction based on baseline cfDNA was used to determine significance. Since MRD positivity is an established poor predictor of the outcome, we assessed the predictability of baseline cfDNA concentration for MRD positivity.

Furthermore, values collected at baseline and follow-up and relapse were also categorized for the two risk groups based on cfDNA ratios, which was calculated as follows:

CfDNA ratio = baseline cfDNA levels/follow-up cfDNA levels.

## Statistics

To examine the ability of our model to predict the outcome for the patient at the time of remission as poor/good by cf-DNA ratios, we applied logistic regression analysis to predict the outcome of patients on follow up based on data available as dead or alive, using cf-DNA ratios and paired t-test for sub group analysis. For analysis, a *p*-value <0.05 was used to suggest a significant relationship (95% confidence interval). A paired *t*-test was applied for cfDNA ratios to predict the outcome for individual patients. All statistics were performed using the R package (Creative Commons Attribution-Share-Alike International License version 4.0).

## Results

Twenty-five patients diagnosed with AML and ALL based on molecular and flow cytometric typing were admitted to the in-patient department. Risk assignment was carried out based on NCI risk stratification, and of these 25 patients, 4 patients were of standard risk (SR) and 1 patient was of IR, while the majority of patients (80%) were HR patients. Of these, four HR patients passed away.

1) Prognosis by concentration of baseline cfDNA

Initially, we decided to predict the outcomes by the concentration of baseline cfDNA, as was done in other solid tumors. A standard reference cut-off was decided based on the published values of cfDNA threshold—180 ng/mL (Phallen et al., 2017).

The groups were divided based on baseline cfDNA concentrations, with 4 patients above the threshold and 17 patients below the threshold, i.e., 180 ng/mL. Of these, all the four high-cfDNA patients were in high-risk groups, but only two patients had poor outcome and two had good outcome. However, higher baseline cfDNA was correlated with stronger MRD positivity (*p*-value 0.32), but the results could be confounded by other predictors for MRD positivity such as higher leukocyte count and poor NCI risk category. However, because of the limited sample size, the results cannot be extrapolated to a larger cohort with statistical precision. Hence, cfDNA concentration at baseline could be used to surrogate MRD prediction in leukemia cases, especially where MRD is not possible or baseline FCM was not carried out. The results for predicting the outcome in high-risk patients with high cfDNA were insignificant (*p*-value 0.32); furthermore, the results for low-cfDNA baseline of high risk were also insignificant (*p*-value 0.92), when predicting relapses and non-responders. We failed to correlate the outcome in the two groups—low-baseline cfDNA group and high-baseline cfDNA group—with the risk criteria and prediction based on baseline cfDNA; hence, larger studies are required to substantiate these findings.

The outcomes given in Table 2 also appear to be governed by the MRD status, which is a known prognostic indicator for outcomes in both the groups. Those patients in the high-risk group were MRD-positive, which determined poor outcomes. We found an association of baseline cfDNA with the MRD status in three patients, of which two had poor outcomes. Hence, cfDNA can prove to be a valid alternative for the prediction of MRD positivity in approximately 75% of cases; the results, however, did not reach statistical significance. Hence, baseline cfDNA can predict, with a certain degree of probability, that the patient might be positive for minimal residual disease, which is an independent prognostic factor.

**TABLE 2:** Prognosis prediction by cfDNA concentration levels at baseline; the outcomes of patients of high risk and other risks (SR and IR) did not correlate with those of high cfDNA concentrations (Phallen et al., 2017) but correlated with MRD positivity.

Category (baseline cfDNA levels >180 ngm.ml*)	Patients above the threshold	High risk	Other risks (IR and SR)	Poor outcome (relapse/resistant disease)	MRD positivity	*p*-value
High baseline cfDNA	4	4	0	2	3 (75%)	0.32
Low baseline cfDNA	21	17	5	3	3 (14%)	0.92

The levels of cfDNA at baseline were also positively correlated with the total leukocyte count levels as cfDNA is a part of tumor fragments and tumor lysis occurring more commonly in patients of higher blast counts. For the patients of the high-risk group, when compared to a similar risk group in low baseline cfDNA levels, relapse or resistance for disease could not be predicted. cfDNA at baseline was, hence, not a significant predictor for the relapse/response of patients directly, but it predicted the MRD positivity for the patient at the time of remission, which is the strongest direct predictor for outcome. When compared to solid tumors based on a higher initial cfDNA concentration, the ranges of cfDNA were wide, and those with a higher cfDNA concentration also had signification log reduction in the remission cfDNA.

2) Prognosis by cfDNA ratios

To explore and analyze our results further, we found the ratios of the cfDNA concentration at two end points of our study—baseline and follow-up (remission or relapse).

The patients were then divided into two groups: a high-cfDNA ratio group and low-cfDNA ratio group. The clinicopathological variables were correlated to assess the risk as per the NCI risk criteria, and then, the ratios were used to predict the prognosis of these patients. We also calculated the statistical power of predicting outcomes (relapse and resistant disease) using the paired t-test in the case of low-cfDNA ratio versus high-cfDNA ratio patients, which was statistically significant (p-value 0.0017), with SD at 2.6. This outcome is mentioned in Table 3.

**Table 3:** Prognosis based on cfDNA ratios (baseline/follow-up cfDNA); the cut-off was decided statistically to be 2.6 (baseline/follow-up cfDNA).

Category	Number of pts	High risk	Other risks	MRD-positive	Poor outcome (relapse/resistant disease)	*p*-value
High cfDNA ratio (>2.6)	8	8 (100%)	0	1 (12.5%)	5 (62%)	0.0017
Low cfDNA ratio (<2.6)	17	12 (70%)	5 (30%)	5 (29.4%)	0	0.002

The overall prediction probability for the ratio was better than the baseline cfDNA levels (p-value 0.0017), suggesting this to be a robust alternative for prognosis prediction in leukemia patients. When comparing the predictability of patients with higher cfDNA ratios, the t-test yielded significant results, with p-value as 0.0017. It was also interesting to note that of all the patients with a high-risk cfDNA ratio, all the patients in the high-risk group passed away, except one patient, who relapsed and is currently on palliative care.

Interestingly, in the high-cfDNA ratio group, the MRD failed to identify relapses because of the eight (12%) patients who had higher ratios of cfDNA, and only one patient had MRD. Even though the low-cfDNA group had five (29%) MRD-positive patients, the outcomes were all favorable as per disease classification, as suggested by our categorization of the low-cfDNA group.

Table 4 highlights logistic regression analysis was also carried out for the group of high cfDNA ratios and outcomes (relapse/resistant disease). The group with high cfDNA ratios had poor outcome prediction if the ratio values were more than 2.6, with significant results (*p*-value 0.03).

**TABLE 4:** Predictive probability of all patients based on cfDNA ratios and their clinicopathological parameters.

Sl. no.	Demographic data		At diagnosis (high cut-off 180 ng/mL)		At the time of remission/relapse		Critical ratios		Risk	Outcome (relapse/resistance)	Notes	Individual predicted probability by cfDNA ratios
	Age (years)	Sex	CfDNA (Cfa)	RNA (Ra)	CfDNA (Cfb)	RNA (Rb)	RNA ratio)	Cf ratios (Cfa/Cfb)				
1	5	M	66.6	144	10.6	122.6	1.174	6.28	HR	Alive	Neg	0.44
2	4.5	M	111	158.2	49.2	311.5	0.507	2.26	HR	Alive	+ve TEL-AML1	0.07
3	2.5	F	160.7	76.8	37.1	50.3	1.526	4.33	HR	Poor, dead	Neg	0.20
4	11	M	29.1	89.2	17.9	103.4	0.862	1.63	HR	Alive	Neg, MRD + ve	0.05
5	4	M	268.9	418.9	28	157.9	2.652	9.60	HR	Poor, dead	MRD + ve	0.83
6	11	F	70.6	766.9	22.3	76.5	10.02	3.17	HR	Alive	Neg	0.12
7	12	M	8.9	112.6	23.1	1451.4	0.077	0.39	HR	Poor, alive	Relapse	0.03
8	13	F	113.5	183.5	17.5	150.3	1.220	6.49	HR	Poor, dead	BM relapse	0.46
9	6	M	154.8	221.7	25	158	1.403	6.19	HR	Alive	consolidation, meningitis	0.42
10	11	M	39.4	1789.5	28.2	252.4	7.089	1.40	HR	Alive	Axillary vein thrombosis, MRD + ve	0.05
11	4	F	15.7	330.3	43.2	43.2	7.645	0.36	HR	Alive	MRD -ve	0.03
12	1.5	F	43.5	947.4	22.2	22.2	42.67	1.96	HR	Alive	MRD + ve, CALLA + ve	0.06
13	6	F	34.2	272.3	26.5	58.1	4.686	1.29	HR	Alive	ATRA syndrome	0.04
14	5	F	40.4	1293.3	53	770.2	1.679	0.76	IR	Alive	CALLA + ve, on induction MRD + ve	0.03
15	2	F	4.7	199.1	19.4	691.6	0.287	0.24	SR	Alive	CALLA + ve	0.02
16	4.5	M	26.8	156.1	30	460.4	0.339	0.89	SR	Alive	CALLA + ve	0.04
17	10	M	97.4	278.6	58.6	204.3	1.363	1.66	HR	Alive	With BCR-ABL + ve MRD + ve	0.05
18	4	M	62.4	47.2	32.7	287.3	0.164	1.91	SR	Alive	CALLA + ve	0.06
19	8	F	79.2	113.8	35.5	63.3	1.797	2.23	SR	Alive	Relapse, CALLA + ve, MRD + ve	0.07
20	7	M	14.3	1738.9	90.1	123.6	14.068	0.16	HR	Alive	CALLA + ve, MRSA + ve	0.02
21	9	M	40.6	919.2	15.5	40.2	22.865	2.62	HR	Dead	Neg	0.09
22	4	F	443.6	1587.4	82.9	741.1	2.142	5.35	HR	Alive	CALLA + ve	0.31
23	5	F	692.3	54.1	114.3	777.1	0.069	6.06	HR	Alive	CALLA + ve	0.40
24	3	F	51.2	490	26.1	727.1	0.673	1.96	HR	Alive	Neg	0.06
25	7	M	3.2	35.1	19.7	371.1	0.094	0.16	HR	Alive	MRD -ve	0.02

The predicative probability of patients based on cfDNA: by statistics, we highlighted that these patients had a higher incidence of relapse, and this was probably independent of the MRD analysis, which is a single independent prognostic factor for the prediction of prognosis. The factors responsible for this could be that if a higher baseline cfDNA was observed in a patient, the same patient, if the prognosis was good, underwent a rapid clearance of tumor cells, and the reduction of cfDNA at remission was to a greater extent than in a patient who had a lower value of cfDNA and did not clear in remission. So, isolated higher cfDNA at diagnosis also had a higher clearance of cfDNA at remission. This could be a responsible factor for patients who did not undergo clearance of tumor, and in turn, cfDNA could be a poor prognostic factor when we compare ratios.

## Discussion

Leukemia, a prevalent cancer in children and a major cancer in adults, involves the unchecked growth of abnormal white blood cells in bone marrow and blood. It remains a significant challenge in oncology, despite progress in molecular insights and treatment strategies. Owing to its diverse causes and bleak outcomes, on-going research seeks novel predictive biomarkers (Alaggio et al., 2022).

Cell-free DNA, also referred to as liquid biopsy, has transformed the landscape of cancer diagnosis and monitoring. It consists of short DNA fragments released into the bloodstream, including those shed by apoptotic or necrotic cells. The presence of cfDNA in human blood was initially recognized by Mandel and Metais in 1948. Subsequently, in 1977, Leon et al. observed significantly elevated cfDNA levels in cancer patients, reaching up to 5,000 ng/mL, in stark contrast to the minimal range of 0–100 ng/mL observed in healthy individuals. Quantitative studies have revealed that healthy subjects typically exhibit cfDNA concentrations between 0 and 100 ng/mL, with an average of approximately 30 ng/mL, while cancer patients display a wider range, from 0 to 1,000 ng/mL, with an average of 180 ng/mL (Bronkhorst et al., 2019; de Martino et al., 2012; Phallen et al., 2017; Cheng et al., 2018). This diagnostic advance offers promising prospects for early detection and disease monitoring in leukemia patients.

The advent of NGS technology has significantly bolstered the utility of cfDNA by enabling the identification of genetic mutations associated with various malignancies. In the realm of solid tumors, cfDNA has emerged as a promising tool, particularly for prognostic purposes. A plethora of studies underscore the significance of baseline cfDNA levels as valuable prognostic indicators in solid tumors, including renal, breast, genitourinary, and brain tumors, showcasing remarkable concordance with tissue samples (Vasioukhin et al., 1994). This non-invasive approach represents a compelling avenue for enhancing cancer diagnosis and monitoring, ultimately advancing patient care. Studies have consistently demonstrated the utility of cfDNA in detecting genetic alterations and monitoring disease progression. For instance, Christina et al. (2017) and Perez-Ramirez et al. (2016) showcased the effectiveness of cfDNA in identifying mutations in epidermal growth factor receptor (EGFR) flow and Kirsten rat sarcoma viral oncogene (KRAS) genes among non-small-cell lung cancer (NSCLC) patients, aiding in treatment decisions. Ren-Hao et al. (2017) and Chan et al. (2021) also illustrated how cfDNA can track the evolution of mutations in colorectal cancer cells, providing insights into therapeutic resistance. Mao li et al. provided quantitative and qualitative evidence in favor of using cfDNA analysis in lymphoma patients for diagnostic and prognostic assessments.

However, the accurate detection of low-level mutations in cfDNA poses challenges. Achieving high sensitivity and specificity, especially for rare mutations, is technically demanding, limiting the clinical utility of cfDNA assays (Krasic et al., 2021). Tumors exhibit genetic heterogeneity, housing various sub-clones, making the comprehensive detection of all relevant mutations in cfDNA challenging and potentially resulting in incomplete or inaccurate results (Lu and Liang, 2016; Greytak et al., 2020; Venetis et al., 2023).

In the context of tumors, cfDNA faces rapid clearance by DNase enzymes in the kidney, liver, and blood and is typically eliminated within 6 h. Conditions such as sepsis, stroke, and fever can increase cfDNA levels due to cell lysis and cellular machinery activation (Lu and Liang, 2016). Technical artifacts or biological factors, such as clonal hematopoiesis, may yield false-positive or false-negative results, complicating cfDNA data interpretation (Leon et al., 1977; Krasic et al., 2021).

Proper collection, handling, and storage of blood samples containing cfDNA are critical as contamination with genomic DNA from white blood cells can dilute the cfDNA signal and compromise mutation detection accuracy (Greytak et al., 2020). Analyzing and interpreting cfDNA data requires bioinformatics expertise to mitigate background noise, and developing standardized, clinically actionable algorithms for data interpretation can be challenging (Bronkhorst et al., 2022).

Furthermore, clinical validation on larger cohorts is essential, demanding dedicated time and resources. cfDNA-based tests must undergo rigorous validation and regulatory approval processes to ensure safety and efficacy before widespread clinical use (Lim et al., 2021).

Extensive research consistently recognizes elevated baseline levels of cfDNA as a robust prognostic indicator across various tumor types, including leukemia. Remarkably, in the context of AML, Mueller et al. (2006) illustrated a correlation between higher baseline cfDNA levels and inferior overall survival, along with an elevated risk of relapse. Nonetheless, our own investigation failed to replicate these findings within our first analysis of cohort, as shown in Table 2, based on cfDNA concentration. It is worth noting that Mueller et al. used nucleosomal DNA, specifically thymidine kinase, in their study involving 25 patients, and, similar to our results regarding the baseline cfDNA concentration, their outcomes did not reach statistical significance. Furthermore, they did not conduct a comparative analysis with MRD, which could potentially act as a confounding variable, a point shown in Table 2. Our findings also suggested that MRD could be predicted with strong suspicion of a higher baseline cfDNA, and in turn, this could serve as a potential surrogate marker for the prediction of the MRD status. Elevated cfDNA concentration and integrity are observed in AML patients compared to healthy controls. ctDNA integrity reflects the MRD status and AML progression, exhibiting a decrease during complete remission (CR) and an increase upon relapse, as demonstrated by Gao et al.

Furthermore, Thierry et al. (2016) emphasized the prognostic significance of elevated cfDNA levels in chronic lymphocytic leukemia (CLL), where patients with elevated cfDNA concentrations exhibited shortened progression-free survival. These findings highlight the broad applicability of cfDNA as a valuable prognostic marker in various cancers, including hematological malignancies such as leukemia. Nevertheless, in the context of chronic lymphocytic leukemia, where WHO risk stratification relies on the total leukocyte count (TLC), this factor could potentially confound their study since patients with a higher TLC also demonstrated higher cfDNA concentrations. Additionally, Anna Rogers suggested the utility of detecting the loss of heterozygosity (LOH) in cfDNA for leukemia prognosis. However, considering resource limitations, comprehensive sequencing of cfDNA may not be feasible for all patients. Alternatively, the clinical application of cfDNA ratios represents a practical approach even in resource-constrained settings.

Based on our observations, a comparison of cfDNA reduction ratios from baseline to the end of induction revealed that patients with ratios exceeding 2.6 experienced post-induction mortality, irrespective of the MRD status in the entire patient cohort. Although our study is limited by a small sample size, both the main and subgroup analyses yielded statistically significant results. Notably, the prediction of leukemia using cfDNA ratio scores demonstrates superior accuracy compared to conventional minimal residual disease detection via flow cytometry, offering a promising advancement in prognosis assessment.

In summary, although cfDNA carries the potential to translate the clinical practice of leukemia in a remarkable way by aiding in the early diagnosis of leukemia by the detection of leukemia-associated mutations and chromosomal abnormalities, providing a non-invasive means of diagnosing the disease where BM is challenging or risky, such as in pediatric patients, Implementing cfDNA-based tests in routine clinical practice can be costly, including the initial investment in equipment, reagents, and operational expenses. There remains a lack of comparability due to inconsistent thresholds. Diagnosing cancer through cfDNA analysis is hindered by the technical hurdles associated with its fragmentary nature and low presence in blood plasma.

On-going research and advancements in technology and standardization efforts will likely play a pivotal role in overcoming these obstacles. As our understanding of the genetic landscape of leukemia expands, cfDNA-based approaches hold promise for improving patient outcomes and advancing leukemia research. Incorporating the clinical applicability of cfDNA into cost-effective methodologies and diagnostic assays holds significant promise, particularly within resource-constrained healthcare settings. This approach can provide substantial benefits to patients, addressing the persistent challenge of leukemia relapse despite the routine availability of other prognostic markers.

## Conclusion

Leukemia remains a complex and challenging hematologic malignancy, with diverse subtypes and underlying genetic alterations. The integration of cfDNA analysis into clinical practice shows potential to revolutionize the diagnosis, monitoring, and management of leukemia, offering new avenues for precision medicine. Despite numerous studies highlighting the role of cfDNA in leukemia, there is a paucity of research specifically investigating the role and significance of cfDNA concentration ratios in predicting prognosis and relapse in acute leukemia patients. This necessitates further investigation to elucidate the potential of cfDNA ratios as valuable biomarkers for improved disease management. cfDNA could be a valuable alternative predictor of the MRD status in leukemia patients as a proof of concept for our study. Our preliminary study involving a cohort of 25 patients successfully validated our initial hypothesis. It proposed a specific cut-off score >2.6 for the ratio of cfDNA at baseline and follow-up for prognostic prediction in these patients, irrespective of the minimal residual disease status. While further investigations are warranted to corroborate our findings, this pilot study presents promising initial results.

## Shortcomings of the study

The study is limited by the small sample size, to be statistically significant to examine all variables predicting prognosis with reference to cfDNA ratios.

cfDNA in tumors is quickly cleared by DNase enzymes in the kidney, liver, and blood, usually within 6 h. Sepsis, stroke, and fever can increase cfDNA levels due to cell lysis and cellular machinery activation. False-positive or false-negative results may occur due to biological factors, such as clonal hematopoiesis. On numerous occasions, the occurrence of false-positive/negative outcomes has been attributed to the presence of clonal hemopoiesis, a condition characterized by the expansion of a single hematopoietic stem cell. Additionally, other significant factors such as the contamination of samples with cfDNA have also yielded similar results. The accuracy of mutation detection can be compromised if blood samples containing cfDNA are contaminated with genomic DNA from white blood cells. Background noise in cfDNA data can be reduced with bioinformatics expertise, but developing standardized algorithms for data interpretation is challenging. Deploying cfDNA-based tests in clinical practice might be expensive. Additional analysis is required to authenticate the results of this pilot investigation with large cohorts and examine the relationship between cfDNA levels and mutational profiles in patients, which could hold significant promise for future clinical applications.

## Data Availability

The original contributions presented in the study are included in the article/Supplementary Material; further inquiries can be directed to the corresponding authors.

## Abbreviations

AML, acute myeloid leukemia; ALL, acute lymphoblastic leukemia; BM, bone marrow; CALLA, common acute lymphoblastic leukemia antigen; CR, complete remission; ctDNA, circulating tumor DNA; cfDNA, circulating free DNA/cell-free DNA; EGFR, epidermal growth factor receptor; HR, high risk; IR, intermediate risk; KRAS, Kirsten rat sarcoma viral oncogene; MRD, minimal residual disease; MRSA, methicillin-resistant *Staphylococcus aureus*; NGS, next-generation sequencing; NSCLC, non-small-cell lung cancer; SR, standard risk; and TLC, total leukocyte count.
